# Epilepsy Care in the Time of COVID-19 Pandemic in Italy: Risk Factors for Seizure Worsening

**DOI:** 10.3389/fneur.2020.00737

**Published:** 2020-07-03

**Authors:** Giovanni Assenza, Jacopo Lanzone, Francesco Brigo, Antonietta Coppola, Giancarlo Di Gennaro, Vincenzo Di Lazzaro, Lorenzo Ricci, Andrea Romigi, Mario Tombini, Oriano Mecarelli

**Affiliations:** ^1^Unit of Neurology, Neurophysiology, Neurobiology, Department of Medicine, University Campus Bio-Medico of Rome, Rome, Italy; ^2^Division of Neurology, “Franz Tappeiner” Hospital, Merano, Italy; ^3^Department of Neurosciences, Reproductive Sciences and Odontostomatology, University Federico II of Naples, Naples, Italy; ^4^Epilepsy Surgery Center, IRCCS NEUROMED, Pozzilli, Italy; ^5^Sleep Medicine Center, IRCCS NEUROMED, Pozzilli, Italy; ^6^Department of Human Neurosciences, “Sapienza” University of Rome, Rome, Italy

**Keywords:** epilepsy, COVID-19, sleep, depression, anxiety

## Abstract

**Objective:** In early 2020, Italy struggled with an unprecedented health emergency related to the COVID-19 pandemic. Medical care of chronic neurological diseases, such as epilepsy, is being sorely neglected. In this national survey, we aimed at understanding the impact of COVID-19 lockdown on the care of people with epilepsy (PwE) and identifying PwE risk factors for seizure worsening to direct telemedicine efforts.

**Methods:** We administered a 48-items online survey (published on April 11, 2020) including socio-demographic, epilepsy-related, and psychometric variables (BDI-II for depression, GAD-7 for anxiety, and PSQI for sleep) to PwE and people without epilepsy (PwoE). Regression analysis identified predictors of seizure worsening.

**Results:** We collected responses from 456 PwE (344 females) and 472 PwoE (347 females). Outpatient examinations of PwE were postponed in 95% of cases. One-third of PwE complained of issues with epilepsy management, but only 71% of them reached the treating physician and solved their problems. PwE had worse depressive and anxiety symptoms (higher BDI-II and GAD-7 scores; *p* < 0.001) than PwoE. Sleep quality was equally compromised in both groups (47 and 42%). Sixty-seven PwE (18%) reported seizure worsening, which was best explained by the number of anti-seizure medications (ASM) of chronic therapy and the severity of sleep disorder.

**Conclusions:** During the current COVID-19 pandemic, a significant percentage of PwE experienced difficulties in follow-up and a seizure number increase, in particular those chronically taking more ASMs and with poor sleep quality. This dramatic experience outlines the urgent need for validation and implementation of telemedicine services for epileptic patients in order to provide regular follow-up.

## Introduction

Italy is facing an unprecedented health emergency represented by the COVID-19 pandemic due to the SARS-CoV-2 virus. The government imposition of quarantines, travel bans, and lockdown throughout the country has been producing the first effects in limiting the spread of the viral infection. The COVID-19 pandemic led to strict measures of isolation (www.apple.com/covid19/mobility) throughout the Italian peninsula. Almost inevitably, isolation is accompanied by either the onset or the worsening of sleep, mood, and anxiety disorders (1). It is also associated with an increased risk of an inadequate level of medical care for chronic disorders, including epilepsy. The social and behavioral consequences of COVID19 lockdown might increase seizure frequency in people with epilepsy (PwE). Furthermore, the COVID-19 viral infection itself can induce a febrile status, which in turn can reduce seizure threshold (2). The industry lockdown hampers anti-seizure medication (ASM) supplies, whereas the reduced care services limited to emergencies make it difficult for PwE to receive regular follow-up and to keep in touch with their treating physicians. To address these difficulties, Italian neurologists are using several communication strategies (e.g., emails, phone, electronic messages, web conference calls, etc.) to maintain contact with their patients, while continuing to manage the increased need for intensive medical assistance.

In this national survey, we aimed at understanding the real impact of COVID-19 breakdown on PwE in Italy by evaluating the seizure frequency, needs, and behaviors of patients in order to identify possible risk factors for seizure worsening and thus to better focus the implementation of telemedicine strategies.

## Materials and Methods

An online survey was created using the free open-access Google^TM^ Forms (https://www.google.com/forms/about/) application. The survey included an informed consent verification, making it possible for those who did not agree with its terms of use to end the survey without further questions. No personally identifiable information was collected, and data were treated according to the European regulation GDPR n. 2016/679. The survey was published on April 11, 2020, on the Facebook webpage of the LICE (Lega Italiana Contro Epilessia, the Italian chapter of the International League Against Epilepsy, ILAE) Foundation, a non-profit organization promoting research, training, and public awareness about epilepsy.

We set a convenience sample of at least 300 PwE. We planned also to include people without epilepsy (PwoE) as a control group. Subjects younger than 18 years old were excluded from the survey since the psychiatric scales used in the questionnaire are validated for adults only. The survey continued until there was a reduction of >90% in the number of daily answers compared to the 1st day of online publication. The survey was closed at 23:59, April 16, 2020.

The questionnaire contained the following sections:

- Introduction with a brief description of the aim of the study
- Informed consent (mandatory)
- Demographic and social data (age, sex, region, educational level, and marital, and working status)
- Changes in working activities during the COVID-19 period
- Anamnesis for depression and anti-depressant therapy
- The assumption of new anti-depressants, anxiolytics, hypno-inducers, or antipsychotics during the COVID-19 period
- Epilepsy-related questions (for PwE group):
  ° seizure frequency during COVID-19 period and pre-COVID-19 period, number and dosages of ASM in chronic therapy, subjective seizure worsening (PwE were asked “Did your epilepsy worsen, with an increased number or severity of seizures, in the lockdown period? Yes or No”), scheduled neurological examinations/outpatient visits and their deferral (if there were any), rising epilepsy-related issues (ASM availability, adverse effects of ASM, anxiety, depression, need to increase therapy), attempts to contact the neurologist, successes in contacting the neurologist, successes in resolving epilepsy-related issues, adherence to the ASM therapy.
- Psychiatric assessment: Beck depression inventory scale II for depressive symptoms (BDI-II), General Anxiety Disorder-7 (GAD-7) for anxiety symptoms, and Pittsburg Sleep Quality Index (PSQI) for sleep.

The BDI-II is a 21-item self-report measure of common depressive symptoms (3). Each item has four possible responses, and higher total scores are indicative of a greater number and severity of depressive symptoms. Scores ranging from 0 to 13 indicate minimal/no symptoms, 14–19 indicate mild depression, 20–28 indicate moderate depression, and 29–63 indicate severe depression.

The GAD-7 is a valid and efficient tool for screening for anxiety symptoms and assessing their severity in clinical practice and research (4). Scores of 5, 10, and 15 represent cut-off scores for mild, moderate, and severe anxiety symptoms, respectively. When used as a screening tool, further evaluation is recommended when the score is 10 or greater.

Subjective quality of sleep was assessed through the PSQI (5), a self-rated questionnaire that assesses sleep quality and its disturbance over 1 month. We used the validated Italian version of PSQI (6) consisting of a 19-items scale, in which responses are given for the last month, that measures sleep disturbances according to seven dimensions: subjective sleep quality (C1), sleep latency (C2), sleep duration (C3), habitual sleep efficiency (C4), sleep disturbances (C5), use of sleep medication (C6), and daytime dysfunctions (C7). The scores from these seven areas were gathered together, creating a global score considered an indicator of relevant sleep disturbances if >5. As PSQI was one of the variables best at predicting seizure worsening in PwE, we performed a principal component analysis, which helps to reduce the dimensionality of data, on PSQI components in order to evaluate which items are responsible of the variation in total PSQI score in our sample.

The social lockdown started on March 11, 2020, in Italy. We therefore defined the COVID-19 period as the 31 days before the questionnaire was published (March 11 to April 10, 2020), whereas the period between February 9 and March 10, 2020, was defined as the pre-COVID-19 period.

Data were automatically stored on a private account and downloaded as a “.csv” text file.

## Statistical Analysis

Data management and statistical analysis were performed in R 3.6.2 using R studio software. Data manipulation was performed in the tidyverse grammar for R.

Normally distributed data were reported as mean ± standard deviation; normality was checked by quantile-quantile plotting. Non-normally distributed data were reported as median and range. The main items of data were subdivided according to the responder's answer to the following questions: “Do you have a diagnosis of epilepsy?” “Did your epilepsy worsen, with an increased number or severity of seizures in the lockdown period?” According to the answers, subjects were divided into PwE and PwoE. The PwE group was further divided into patients experiencing worsening (WPwE) and those without worsening (nWPwE). This subjective variable was chosen to merge qualitative (intensity, duration, post-ictal symptoms) and quantitative (number) changes to seizures in PwE. However, to check for possible biases caused by the subjectivity of the PwE evaluation of seizure worsening, we compared the seizure frequency changes between WPwE and nWPwe in terms of both absolute number and percentage change (percentage change of seizure frequency in the COVID-19 period with respect to the pre-COVID-19 period).

Differences among groups were described with a *t*-test for continuous normal variables, the *U*-Mann-Whitney test for non-normal continuous variables, and Chi-squared for frequencies. Significant correlations (Pearson's correlation for normally distributed variables, Spearman's correlation for non-normally distributed variables) were calculated for psychometric variables in the PwE and PwoE groups. As, in Italy, the number of COVID-19 infections was higher in the Northern regions of Lombardia, Veneto, and Piemonte (www.salute.gov.it), we also performed a comparison between participants coming from those regions and those resident in the rest of the peninsula.

In order to better describe which variables influenced the worsening of epilepsy in PwE during the lockdown period, we designed a logistic regression model. We selected dependent variables through a stepwise backward model, with selection based on the Akaike information criterion (AIC), which was implemented using the MASS library for R. Categorical variables were dummy-coded prior to entering the logistic regression. Variables in the initial entry were chosen among those showing potential differences between the worsened and non-worsened group (*p* < 0.1). We added to these variables some factors that, in our experience, could increase the likelihood of worsening, such as number of ASMs or previous diagnosis of depressive disorders.

Since PSQI showed to be a significant predictor of seizure worsening, we investigated which component of PSQI contributed the most to the variability of the total score. To better describe the PSQI sub-scores, we performed a principal component analysis (PCA) to reduce the dimensionality of data, choosing components that explained most of the variability (elbow method) and showing which sub-items weighed the most in those components.

The alpha level was set at 0.05 for statistical significance.

## Results

A total of 953 subjects accessed the online survey, and 928 of them (97.4%) gave their consent to proceed with the questionnaire.

### Demographic Data

We collected the answers of 456 PwE (344 females) and 472 PwoE (347 females). Females represented 78% of the whole sample. PwoE were older than PwE (43.9 ± 12.3 years, range 18–89 years vs. 37.82 ± 12.48, range 18–86 years; *p* < 0.0001). Regional residence frequency was, in alphabetical order: Abruzzo 24, Basilicata 3, Calabria 16, Campania 123, Emilia-Romagna 53, Friuli venezia giulia 20, Lazio 315, Liguria 9, Lombardia 112, Marche 11, Molise 10, Piemonte 48, Puglia 49, Sardegna 16, Sicilia 16, Toscana 30, Trentino alto adige 19, Umbria 9, Valle d'aosta 0, and Veneto 42.

We did not find any difference between responders from the regions of Lombardia, Veneto and Piemonte and those from other Italian regions with regard to age, sex, and number of PwE and PwoE.

### Socio-Demographic and Occupational Data

The socio-demographic and occupational data are summarized in Table 1.

**Table 1:** Socio-demographic data.

**Variable**		**PwE**		**PwoE**		**Stat. sig**.
		***N***	**%**	***N***	**%**	
Total		456	100	472	100	
Females		344	75.4	347	79.9	0.6263
Age (mean ± *SD*)		37.9 ± 12.5		42,3 ± 12,32		**<** **0.0001**
Marital status						**0.0001**
	Single	189	41.4	112	23.7	
	Married	164	36.0	224	47.5	
	Cohabiting	77	47.0	93	19.7	
	Separated/Divorced	25	5.5	38	8.1	
	Widowed	1	0.6	5	1.1	
Education						**0.0001**
	Primary school	12	2.6	2	0.4	
	Secondary school	69	15.1	30	6.4	
	High School	211	46.3	167	35.4	
	Bachelors	131	28.7	189	40.0	
	PhD/residency	26	5.7	82	17.4	
	No answer	7	1.5	2	0.4	
Working status						** <0.0001**
	Employee	198	43.4	276	58.5	
	Unemployed	147	32.2	74	15.7	
	Freelancer	56	12.3	83	17.6	
	Retired	45	9.9	32	6.8	
	Laid off	10	7.6	7	1.5	
Job reduction during COVID-19 period						**<0.0001**
	No job reduction	119	45.9	42	16.5	
	Laid off	42	16.2	46	18.1	
	Forced private job shutdown	30	11.6	60	23.6	
	Forced holidays	15	5.8	38	15.0	
	Fired	8	3.1	3	1.2	
	Job hours reduction	45	17.4	65	25.6	
Depression						>0.3
	Positive history	89	19.5	80	16.9	
	Use of anti-depressants at the moment of the survey	35	7.7	39	8.3	
COVID-19 symptoms						>0.06
	Fever	30	6.6	30	6.3	
	Swab sample	16	3.5	29	6.1	
	Positive swab	1	0.2	2	0.4	
	Hospitalization for COVID-19	0	0	1	0.2	

*Bold is used for significant values*.

*SD, standard deviation*.

PwE had a lower percentage of occupation (55.7%, employees + freelancers) than PwoE (76.1% employees+freelancers); 54% of PwE and 83.5% of PwoE reported a reduction in their working activities during COVID-19 lockdown (Table 1). Of all respondents, 79.7% reported that they stayed at home around the clock.

### Depression

A history of depression was reported by 89 PwE (19%) and 80 PwoE (17%), with 35 PwE (8%) and 39 PwoE (8%) taking anti-depressant drugs at the moment of the survey.

Furthermore, 44 PwE and 32 PwoE reported that since the COVID-19 isolation began, they had started taking new psychotropic drugs (anxiolytics 46.5%, anti-depressants 8.5%, antipsychotics 2.8%, and hypnotics 42.3%) for insomnia (38.2%), depression (14.5%), and anxiety (47.4%).

### SARS-CoV-2 Infection

Symptoms of COVID-19 infection was specifically investigated. It was found that 32 PwE reported fever (6.6%) and 16 PwE (3.5%) underwent a nasopharingeal swab test for *SARS-CoV-2* (one positive, 0.2%, no hospitalization was required), while 30 PwoE (5.3%) had fever and 29 (6.1%) underwent the swab test (two positives, 0.4%, one with hospitalization, one with spontaneous recovery). No significant differences between PwE and PwoE were found.

### Psychiatric Questionnaires

#### Depressive symptoms

Overall, PwE had more sever depressive symptoms (higher BDI-II scores) than PwoE (PwE 12.0 ± 10, PwoE 9.8 ± 7.7; *p* = 0.0001).

Among PwE, 297 subjects (65.1%) had normal BDI-II values (score ≤ 13), 69 (15.1%) had mild depressive symptoms (14–19 score), 53 (11.6%) had moderate depressive symptoms (20–28 scores), and 37 (8.1%) had severe depressive symptoms (>28 score). In PwoE, 357 subjects (75.6%) had normal BDI-II values, 64 (13.6%) had mild depressive symptoms, 33 (7%) had moderate depressive symptoms, and 18 (3.8%) had severe depressive symptoms.

No difference was found between responders from the regions of Lombardia, Veneto, and Piemonte (the areas most severely affected by the outbreak) and those from other Italian regions.

#### Anxiety symptoms

PwE had more severe anxiety symptoms (higher GAD-7 scale scores) than PwoE (8 ± 5.3 and 6.8 ± 4.9, respectively; *p* = 0.0002).

Among PwoE, 135 (47.5%) had normal GAD-7 values, 128 cases (32.4%) had mild anxiety symptoms, 142 (12.9%) had moderate anxiety symptoms, and 34 (7.2%) had severe anxiety symptoms.

Among PwE, 111 (39.5%) had normal values, 92 cases (28.9%) had mild anxiety symptoms, 162 (21.3%) had moderate anxiety symptoms, and 47 (10.3%) had severe anxiety symptoms.

No difference was found between the GAD-7 values of responders from the regions of Lombardia, Veneto, and Piemonte, and those from other Italian regions.

#### Sleep quality

PSQI scale scores did not differ between PwE and PwoE (6.8 ± 3.7 and 6.6 ± 3.8, respectively; *p* = 0.3117), and 214 PwE (46.9%) and 200 PwoE (42.4%) had PSQI values out of normal range (>5).

The first three components of the PSQI explained 75% of the variance of the total PSQI score. The sub-items explaining most of the variance of total PSQI were, in order of relevance: C1: Subjective evaluation of sleep quality; C2: Sleep Latency; C3: Sleep duration. Full PCA data and weights are shown in Table S1.

No difference was found in sleep quality between responders from the regions of Lombardia, Veneto, and Piemonte, and those from other Italian regions.

#### Epilepsy

##### Seizures in PwE

PwE reported having generalized epilepsy in 188 cases (41.2%) and focal epilepsy in 139 cases (30.5%), whereas the remaining 129 PwE (28.3%) were not aware of the epilepsy type they suffered from. Among the whole sample of PwE, 212 (46%) had been free of seizure in the last year.

In non-seizure-free patients, PwE reported to take a median of 2 ASMs (range 0–7) and had a median of 1 seizure (range 0–100) in the pre-COVID-19 period, and a median of 1 seizure during the COVID-19 period (0–100; Wilcoxon signed ranks test Z−1-138, asympt sign. 0.255).

Sixty-two patients (13%) reported that they had experienced at least one generalized tonic-clonic seizure during the COVID-19 lockdown.

We did not find any difference between responders from the regions of Lombardia, Veneto, and Piemonte, and those from other Italian regions with regard to number of seizures.

##### Worsening of seizures

**Figure 1:**
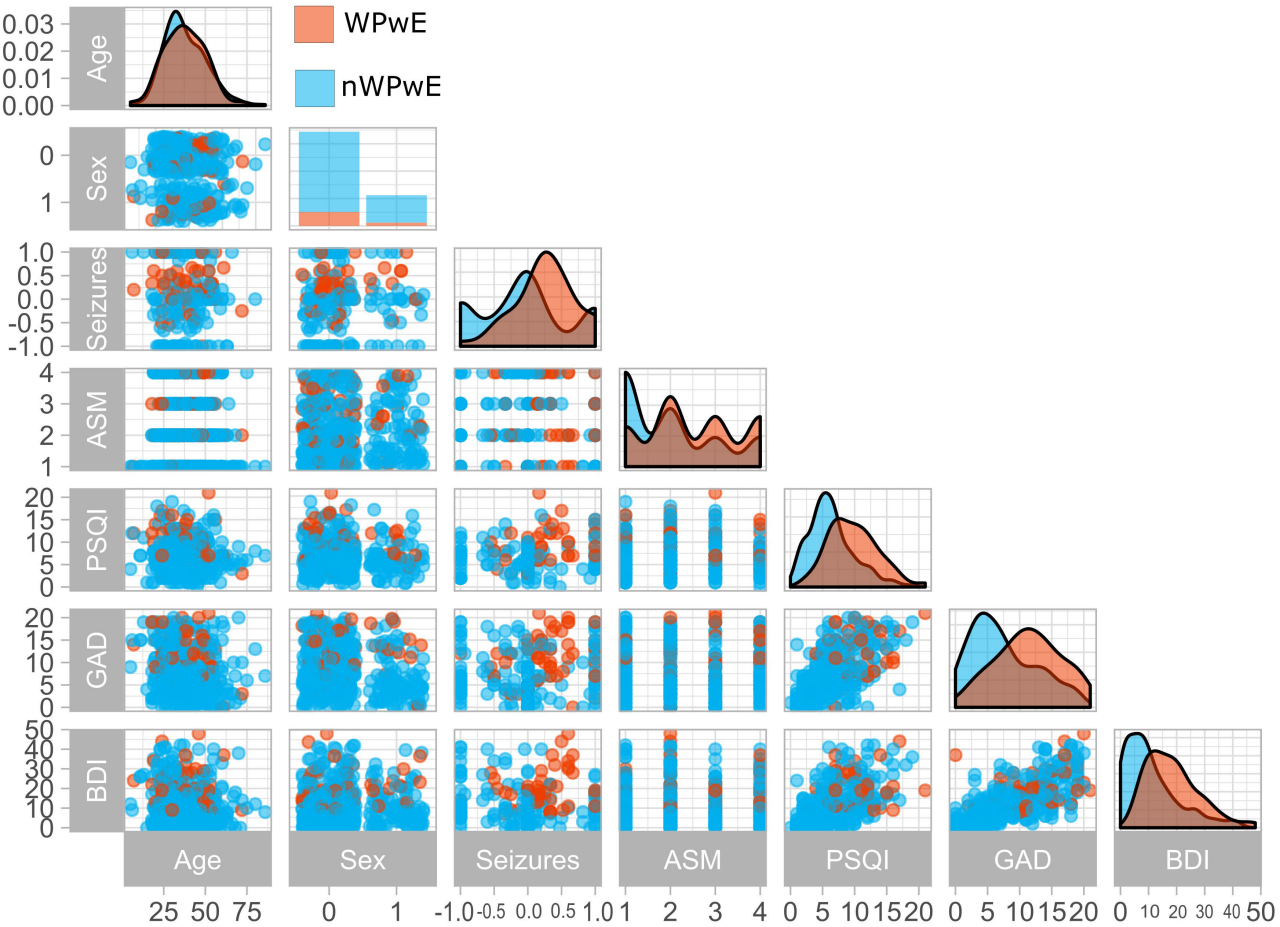
Clinical variables in people with epilepsy according to seizure worsening during the COVID-19 pandemic. The figure displays a paired plot that highlights differences between PwE who experienced worsening (Orange) and those who did not (Blue). WPwE, Worsened People with Epilepsy; nWPwE, non-Worsened People with Epilepsy; ASM, Anti-Seizure Medications; GAD, Generalized Anxiety Disorder 7; PSQI, Pittsburgh Sleep Quality Index; BDI, Beck Depression Inventory; Sex (0 = Female,1 = Male); Seizures (Percentage increase/decrease in seizure frequency compared to previous months).

A worsening of seizures during the COVID-19 period was reported by 67 (18%) PwE (WPwE). In WPwE, but not in not-worsening PwE (nWPwE), the number of seizures was higher in the COVID-19 period (median 1, range 0–50) compared to the pre-COVID-19 period (median 3, range 0–80; *p* = 0.0003). WPwE had a percentual worsening of seizure frequency, with a median value of 25% (0–60%) (Figure 1). Compared with nWPwE, WPwE more frequently had a positive history of depression (*p* = 0.01), anti-depressant use (*p* < 0.0001), tonic-clonic seizures during COVID-19 (*p* < 0.0001), and epilepsy-related issues during COVID-19 (*p* < 0.0001); conversely, seizure freedom was less frequent in WPwE (*p* < 0.0001). In the psychiatric questionnaires, WPwE had more severe depression and anxiety symptoms and more disturbed sleep than the rest of the PwE group (*p* < 0.001).

#### Improvement of Seizures

We identified 61 PwE (13%) patients that, according to their self-reported seizure frequency, had improved during the lockdown period. These PwE had an average reduction of seizures according to their self-report of 4.8 ± 4 seizures/month, with an average percentage improvement in seizure frequency of 72 ± 31%.

We compared their psychometric scores with those of the WPwE group.

No significant difference was found in total BDI scores between the two groups (WPwE: BDI = 17.69 ± 9.99; improved PwE: BDI = 15.25 ± 12.02; *T* = 1.23, *p* = 0.216), and PSQI total score was significantly lower (better quality of sleep) in improved PwE (WPwE: PSQI = 9.4 ± 4; Improved PwE: PSQI = 6.59 ± 3.17; *T* = 3.8 *p* = 0.00002). Anxiety symptoms were less severe (lower GAD scores) in improved PwE (WPwE: GAD = 10.84 ± 5.4; improved PwE: GAD = 8.69 ± 5.48; *T* = 2.23, *p* = 0.02).

##### Epilepsy-related issues during COVID-19

**Figure 2:**
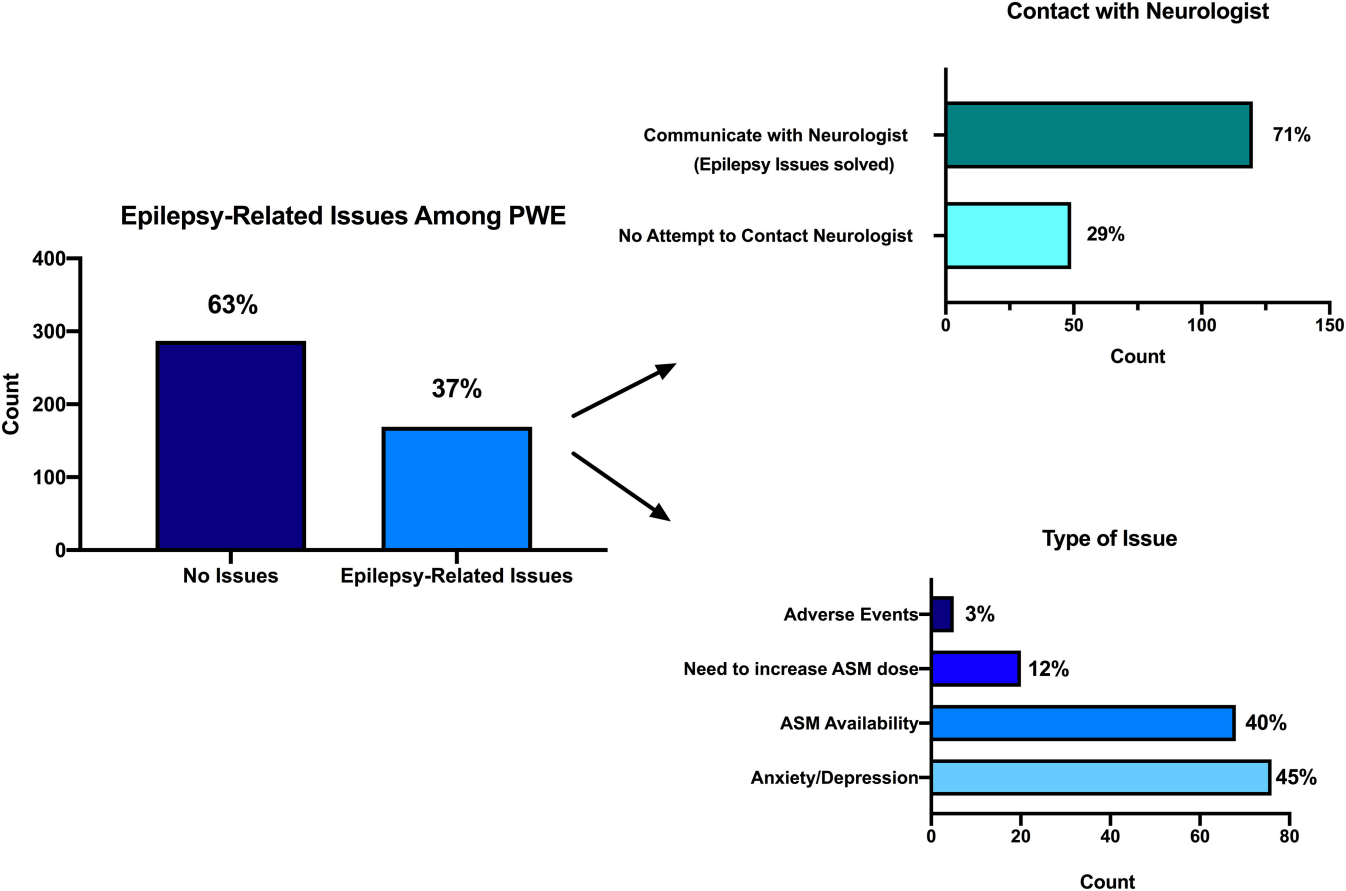
Epilepsy-related issues during the COVID-19 pandemic. Among the whole PwE sample, 169 (37%, in this figure) persons reported negative issues related to the management of epilepsy (left). Attempt to contact treating neurologist (right up): 120 (71%) out of 169 PwE managed to reach their neurologist, and all of them solved their problem thanks to the advice given by the treating physician. Types of issues they were concerned about (right down): 68 PwE (40%) had problems with ASM availability, 20 PwE (12%) had to increase their therapy, 6 (3%) experienced ASM-related adverse effects, and 75 (44%) had anxiety/mood problems.

Of PwE, 172 (38%) reported to have a scheduled outpatient visit during the COVID-19 period; 166 of them (96%) did not receive it. Among the whole PwE sample, 169 (37%, Figure 2) persons reported negative issues related to the management of epilepsy (61% of these patients had a planned examination that was deferred). Also, 68 PwE (40%) had problems with ASM availability, 20 PwE (12%) had to increase their therapy, six (3%) experienced ASM-related adverse effects, and 75 (44%) had anxiety/mood problems. All of these subjects attempted to contact the treating neurologist using short text message (SMS)/WhatsApp messages (43%), emails (25%), personal mobile calls (22%), or direct doctor's office calls (9%). Of 169 PwE, 120 (71%) managed to reach their neurologist, and all of them solved their problem thanks to the advice given by the treating physician. No PwE patient was hospitalized for epilepsy-related problems. PwE complaining of problems with ASM availability were taking the eight ASM molecules carbamazepine, clonazepam, eslicarbazepine acetate, lamotrigine, levetiracetam, phenobarbital, valproic acid, and zonisamide, either alone, or in different combinations.

##### Therapy compliance

Of PwE, 424 (93%) reported having taken their ASMs regularly during the COVID-19 period, whereas 32 (7%) reported inadequate adherence due to forgetfulness (70%), demotivation (15%), adverse events (10%), or difficulties in ASM supply (5%). No PwE reported low adherence to ASM therapy because of a difficulty in contacting the referring physician, though 94 PwE (22%) complained of issues in the retrieval of ASMs. Also, 13 (2.6%) patients had a vagus nerve stimulator; three (23%) of them had problems with their device, and these were solved by contacting their neurologists.

##### Correlations

We performed a correlation analysis among the three psychometric scales, age, number of seizures, and variation in seizures between the pre-COVID-19 period and the COVID-19 period.

**Figure 3:**
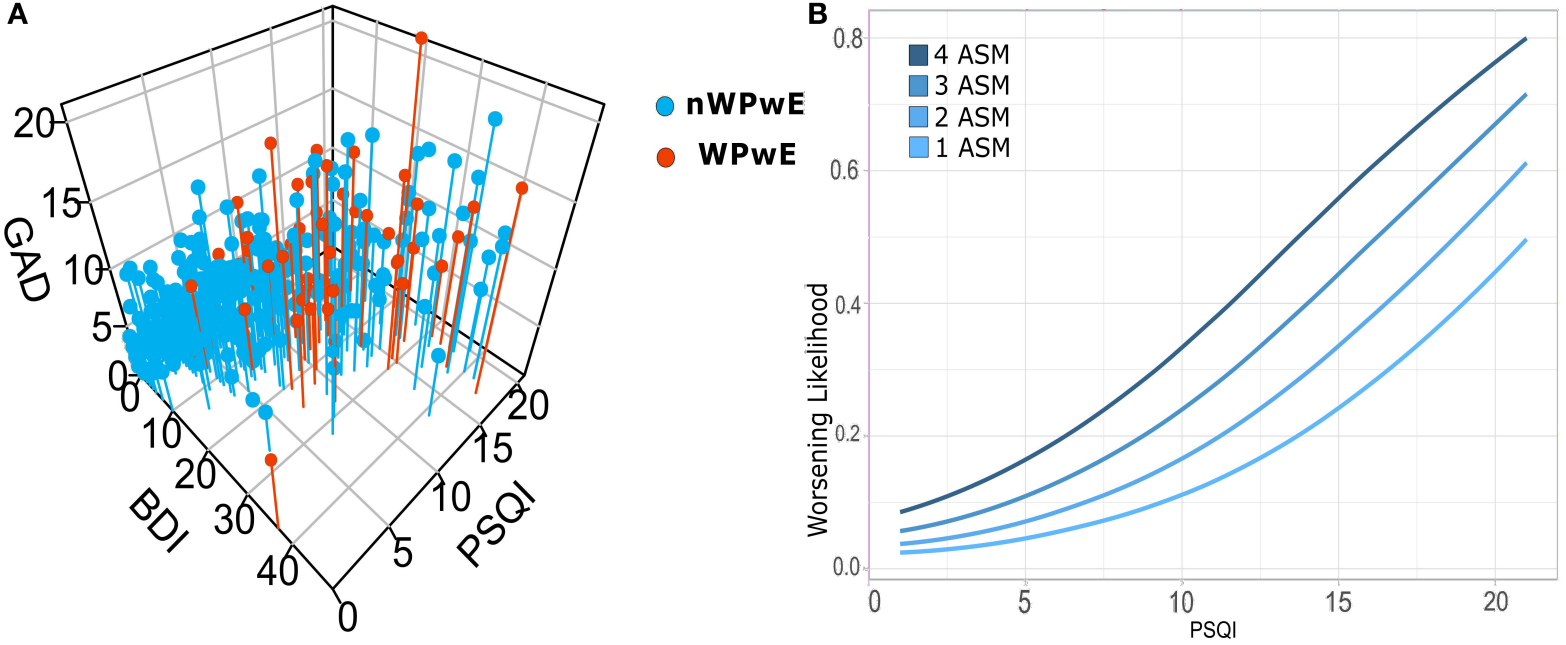
**(A)** Correlations among psychiatric scales for depression (BDI), anxiety (GAD7), and sleep (PSQI), and logistic regression for seizure worsening in PwE. Illustrates the relationship among psychometric (PSQI, GAD, BDI) tests in people with epilepsy; subjects that underwent worsening (WPwE) are color-coded (red). In the 3D plot, it can be noted that WPwE tend to fall within a widespread cluster of subjects with more impairment, while there is a dense cluster of subjects with normal tests that do not report worsening. **(B)** In order to evaluate which psychometric test and variable did independently influence the likelihood of worsening, we designed a logistic regression model that identified the number of ASMs and the PSQI score as significant predictors. We show the increase in the likelihood of worsening for each score of PSQI and for each number of ASMs. WPwE, Worsened People with Epilepsy; nWPwE, non-Worsened People with Epilepsy; ASM, Anti-Seizure Medications; GAD, Generalized Anxiety Disorder 7; PSQI, Pittsburgh Sleep Quality Index; BDI, Beck Depression Inventory.

BDI-II, GAD-7, and PSQI were highly correlated with each other (*r* > 0.530, *p* = 0.0001; Figure 3) and with the presence of tonic-clonic seizures (rho > 0.160; *p* ≤ 0.001).

Age was negatively correlated with BDI-II and GAD-7 (*r* = −0.119, *p* = 0. 011; *r* = −0.148, *p* = 0.002).

The number of ASMs was correlated with the number of seizures either in the pre-COVID-19 (rho = 0.332, *p* = 0.0001) or during the COVID-19 period (rho = 0.238, *p* = 0.0001).

## Logistic Regression

Logistic regression was performed to ascertain the effects on the likelihood of seizure worsening (WPwE) dependent on the following variables: “Age,” “Sex,” “GAD7,” “BDI,” “PSQI,” “Change in work condition,” “History of depression,” “Number of ASMs,” and “Number of seizures in the pre-COVID-19” (Table 2). The variables were introduced into the model with a stepwise backward method, with choice based on the Akaike information criterion (AIC). The logistic regression model was statistically significant for the only two variables surviving after stepwise selection: Number of ASMs and PSQI (Figure 3). Number of ASMs: odds ratio = 1.58 (95% C.I.= 1.12–2.2), Standard Error = 0.17, *z*-value = −2.67, *p* = 0.001; PSQI: odds ratio = 1.20 (95% C.I. 1.10–1.30), Standard Error = 0.04, *z*-value = 4.09, *p* = 0.0001. The model showed an accuracy of 87% in predicting seizure worsening.

**Table 2:** Factors influencing seizure worsening.

**Features**	**WPwE**	**NWPwE**	**Predictor (OR)**
Age	37.6 ± 12	41.19 ± 12	n.s.
Sex	54 F; 13 M	290 F; 54 M	n.s.
GAD-7	10.8 ± 5.4	7.5 ± 5.12	n.s.
BDI-II	17.69 ± 9.99	10.99 ± 9.64	n.s.
PSQI	9.40 ± 4.07	6.40 ± 3.52	**1.20**
Work situation change	41 Yes; 26 No	260 Yes; 84 No	n.s.
History of depression	9 Yes; 58 No	26 Yes; 363 No	n.s.
Number ASM	2.5 ± 1	1.9 ± 1	**1.58**
N. Seizures/month pre-COVID-19	5.18 ± 4	2.26 ± 4	n.s.

*ASM, anti-seizure medication; BDI-II, Beck Depression Inventory-II; PSQI, Pittsburgh Sleep Quality Index; n.s., not significant. Bold values are for statistically significant variables surviving after stepwise selection during the logistic regression*.

## Discussion

Our survey was conducted during the most severe phase of governmental restrictions on mobility, work, and public services caused by the COVID-19 pandemic in Italy. We aimed at investigating the impact of the lockdown on PwE care, identifying epilepsy-specific issues that they had to struggle with.

Epilepsy is a common chronic neurologic disorder, and PwE (particularly those with drug-resistance) need long-term care for managing ASMs, adverse drug effects, and possible behavioral disturbances. In our sample, more than one-third of PwE had a planned outpatient evaluation during the COVID-19 lockdown, and this was postponed in almost all cases. Furthermore, one in three PwE of the whole sample complained of epilepsy-related problems requiring a neurologist's intervention. All of these PwE tried to reach the treating neurologist by phone, messaging, and emails, but only three out of four succeeded. When the PwE achieved contact with the treating neurologist, they always solved their epilepsy-related concerns. Remarkably, no PwE were hospitalized for epilepsy-related problems.

Present data accurately reflect the main features of epilepsy medical care in Italy. Our data confirm the strict personal contact that Italian neurologists have with their patients, which often goes beyond what it is merely due by public health assistance. Furthermore, our data strongly emphasize the need to improve and implement organized telemedicine assistance in the public health system, because in most cases, a simple phone/message/email could resolve medical issues, avoiding unnecessary emergency room, or outpatient examinations. A telemedicine program appears of utmost importance in this historical moment due to the governmental restrictions caused by pandemic but also for the so-called “phase 2” of the pandemic, when restrictions will be progressively waived. Reducing outpatient examinations could decrease the number of in-hospital contacts, minimizing preventable COVID-19 contagions among physicians, and patients themselves, and contributing to the efficiency of public medical assistance throughout the emergency period.

Our survey also suggests that a large part of the medical care for PwE could be effectively managed through telemedicine, particularly for stable patients or those with lower risk of seizure aggravation.

Among PwE, almost one in five reported seizure worsening during the COVID-19 period.

The rate of seizure worsening in PwE reported here is consistent with a small body of previous evidence exploring the impact of environmental stressors on seizure occurrence. Specifically, a retrospective study investigated the frequency of seizures during the Persian Gulf War in 1991 and found that about 10% (eight out of 82 PwE) reported increased frequency (7). During flood evacuation in the Netherlands in 1995, eight out of 30 (26%) interviewed PwE reported seizure worsening (8).

In our sample, we showed that most of the WPwE tended to have severe depressive, anxiety, and sleep disturbance symptoms, while PwE with scores in the normal range were less likely to experience worsening. To investigate the most relevant risk factors that influenced worsening, we focused on socio-demographic and clinical variables and psychometric factors. Logistic regression showed that the number of chronic ASMs and the score of sleep quality (PSQI) significantly influenced the likelihood of worsening. Remarkably, the number of ASMs taken was even superior to the seizure frequency in predicting worsening; this probably reflects a concomitant drug-resistance. With the aim of developing an efficient telemedicine program, the number of ASMs is a more objective parameter assess compared to seizure frequency [which is sometimes difficult to assess objectively and accurately by patients and caregivers (9)]. In addition, the number of ASMs may represent the expression of an overtreatment condition, where patients are taking unnecessary high doses of combination therapy (polypharmacy), and polytherapy-itself may increase side effects and lower adherence, inducing seizure worsening in a vicious circle (10, 11). The analysis we conducted on sleep quality demonstrated that the reduction of sleep time and, generally, insomnia-related issues were the factors most influencing the reported sleep concerns. Interestingly, sleep disturbances did not differ between PwE and PwoE, and abnormal values of PSQI were found in almost half of the subjects, a finding that is consistent with results from general population studies (12). Poor sleep quality could be related to the sudden changes in lifestyle that affected both PwE and PwoE during the lockdown (13). Sleep quality deterioration is often related to depressive (14) and anxiety symptoms (15), but these factors did not enter our regression model. Even if PwE complained of more severe depressive and anxiety symptoms than PwoE, these features did not influence the probability of seizure worsening according to our model. This pronounced effect of sleep is probably related to the strong pathophysiological connection that sleep shares with epilepsy. The bidirectional interactions between sleep, epilepsy, and ASMs are well-known; however, recently, several authors highlighted the rhythmic patterns of epileptic seizures and EEG discharges related to vigilance states and circadian variation in excitatory and inhibitory balance (16, 17). The overall impairment of the sleep-wake cycle due to the COVID-19 emergency may affect both PwE and PwoE; however, sleep fragmentation and sleep deprivation may induce in PwE an increase of EEG epileptiform abnormalities (18) and seizure worsening even through an increase in cortical excitability (19).

It is worth reporting that, in our sample, there was also a small portion of PwE who reported a reduction of seizure frequency and a better sleep quality rather than a worsening. This likely reflects the reduction of chronic working and familial load and furtherly confirms the beneficial effect of sleep on seizure control.

### Other Epilepsy-Related Negative Issues During the COVID-19 Pandemic

Anxiety and depression symptoms were identified as the most frequent epilepsy-related issues reported by PwE during the COVID-19 pandemic. Population-based studies demonstrated that one in every three PwE experiences a psychiatric disorder in the course of life, with mood and anxiety disorders being the most frequently documented comorbidities in adults and children (20, 21). It was expected that in a condition of stress, as the COVID-19 isolation surely is, both depression and anxiety would worsen. Furthermore, their exacerbation could have also been favored by the concurrent seasonal changes, which are factors aggravating psychiatric disorders in people with depression and anxiety (22). Depressive symptoms in PwE are known to correlate with seizure frequency and intensity (21). However, we assessed the depressive and anxiety symptoms only during the COVID-19 period and not in the pre-COVID-19 period, so we cannot discuss modifications induced by the COVID-19 pandemic.

The second most relevant epilepsy-related issue of PwE during COVID-19 was ASM availability. At the time of the survey, there was no advice on the Italian Medicines Agency (Agenzia Italiana del Farmaco, AIFA) website about the lack of availability of ASMs in Italy during the COVID-19 period. However, in March, the LICE website published a note about the reduced availability of one ASM (valproic acid), which was caused by production problems at the pharmaceutical company. However, the problems related to drug availability involved eight different ASM molecules and were not restricted to valproic acid alone.

Adverse events and necessity of increasing the dosage of ASMs were the other two epilepsy-related problems reported.

Among our PwE, only 7% reported reduced compliance with ASM treatment; this number is quite low compared to data on self-reported non-adherence available in the literature [for a review, see (23)]. We can hypothesize that home isolation may have favored compliance for therapy due to a lack of possible distracting factors, such as work, school, or recreational and leisure activities.

### COVID-19 Infections

Among our responders, we found a 0.2–0.4% rate of positivity to SARS-CoV-2 virus infection. This corresponds perfectly to the Italian national trend (152,271 COVID-19 positive cases by April 11, 2020 (data provided by the Minister of Health, www.salute.gov.it), in an Italian population of 5,9433,744 (data from the ISTAT, the National Institute of Statistics, www.istat.it 2011 census), with a resultant 0.2% rate of infection). We did not find any difference in the number of contagions between PwE and PwoE, thus supporting the notion that PwE do not have a higher risk of contagion with respect to the general population.

### Differences Between PwE and PwoE

In our sample, we found some socio-demographic differences between PwE and PwoE, which are consistent with those previously described in the literature. In particular, PwE had a lower educational level, were less occupied, and less likely to be married/cohabiting than PwoE. These are factors depending either on epilepsy itself (unpredictability of seizures, impairment of consciousness during seizures, cognitive, and motor disability, behavioral problems) and its consequences (e.g., driving limitations) or on epilepsy-related stigma (24).

The consistency of these socio-demographic data, but also those regarding drug-resistance (25) and psychometric findings, reveal that our sample is accurately representative of the entire epileptic population.

### Limitations

We received answers that were unbalanced for sex, with a clear predominance of females. However, our percentage of female respondents was quite similar to the proportion of female users on the Facebook social network in Italy (female:male ratio 2:1, https://wearesocial.com/it/digital-2020-italia). While for depressive symptoms, there is no gender prevalence in either the general population (26) or PwE (21), anxiety symptoms are more frequent in females in the general population (27) but not in PwE (28). Sleep quality does not have a gender prevalence in PwE (29), while, in the general population, sleep disturbances seem to be more prevalent in males (30). Thus, anxiety and sleep quality scores in the present manuscript could be influenced by the unbalanced distribution of genders, but the proportion of females was not different among PwE and PwoE, so it is unlikely that it could affect differences among these groups.

Secondly, most of our responders were young adults, and the sample could not therefore be representative of the general prevalence of epilepsy across the entire lifespan (31). However, it probably reflects the age distribution of people more acquainted with the use of the Internet (32), which was the channel of distribution of our questionnaire. We are aware that the use of this channel introduced several biases, but our scope was to reach the highest number of PwE nationwide during the lockdown phase of maximal restrictions and, thus, the Internet offered the best opportunities to achieve our goal. Thus, our data could not reflect those of persons most affected by the SARS-CoV-2 virus (older than 65 years) and may underestimate the impact of the viral infection on PwE. However, the rate of COVID-19 positivity in our sample fitted the Italian mean of contagions. Furthermore, our principal aim was to explore the social and medical consequences of the COVID-19 lockdown on PwE epilepsy management and not how the viral infection influenced the epilepsy symptoms. The reduced number of elderly people could also bias the number of reported seizures, drug-resistance rate, and consequently, number of anti-seizure medications, which are often lower in this portion of the population. The use of the Internet for questionnaire diffusion did not allow PwE with moderate-severe cognitive impairment, who represent a relevant subpopulation in PwE, to participate, and thus this type of PwE was not explored at all by our survey.

A further limitation of our study was that we did not execute an analysis of differences between generalized and focal epilepsies. We chose to not perform this analysis for two main reasons: about one in three patients declared that they did not know the classification of their own epilepsy, so we are conscious that the reliability of the reported diagnosis could be very low. In fact, the differential diagnosis between generalized and focal epilepsy is sometimes a difficult issue also for a neurologist/epileptologist.

In our survey, only 2.6% of patients had a vagus nerve stimulator; this reflects the more severe cognitive impairment that can often affect PwE, and this can explain the low number of these patients participating in the survey.

Our sample was not equally distributed across the country, with a higher contribution of PwE coming from the Lazio region, which was not the region with the highest number of COVID-19 cases. However, the aim of our study was to explore the scenario of social isolation induced by the “lockdown” and its influence on epilepsy care. In this regard, it is important to note that governmental restrictions on mobility and access to health services were homogeneous throughout the country, in particular in the 1st month of the lockdown, when our survey was conducted. Furthermore, none of our epilepsy-related and psychometric scales were different when comparing the values of the regions with the highest number of contagions with the rest of Italy.

Another limitation of the study is that it does not provide data on the impact of lockdown on depressive and anxiety symptoms because they were assessed only during the lockdown, without any question on the preceding period. However, we designed to use psychometric scales only as covariates to understand their contribution to seizure modifications and not as the main outcome variable of our study on lockdown impact on PwE.

We are aware that an observational online survey provides low strength of scientific evidence. However, the global lockdown did not allow any other kind of contact with our patients, except for the emergency room. Thus, we decided to promote this survey to understand the real and current needs of our PwE to improve the implementation of remote medical assistance in epilepsy.

## Conclusions

The enormous and unprecedented social restrictions caused by the COVID-19 pandemic really put the Italian National Health System to the test and negatively impacted medical care for epilepsy. PwE struggled with difficulties in follow-up and attempted to reach their doctors in many different ways for answers to their complaints but did not always succeed. The number of chronically taken ASMs and sleep deterioration were the major factors influencing the risk of seizure worsening experienced by some patients. Special attention should be paid to these factors to prevent seizure worsening in PwE and to help set up an efficient telemedicine program devoted to epilepsy care.

## Data Availability Statement

The data analyzed in this study is subject to the following licenses/restrictions: The dataset will be provided to reviewers if required. Requests to access these datasets should be directed to g.assenza@unicampus.it.

## Ethics Statement

Ethical review and approval was not required for the study on human participants in accordance with the local legislation and institutional requirements. Written informed consent was implied via the completion of the questionnaire.

## Author Contributions

GA: design and conceptualized study. JL: major role in methods creation, statistical analysis, and interpretation. FB: major role in statistical analysis. AC, GD, AR, and MT: major role in acquisition of data. VD and OM: interpreted the data and revised the manuscript for intellectual content. LR: major role in iconographic creation. All authors contributed to the article and approved the submitted version.

## Conflict of Interest

The authors declare that the research was conducted in the absence of any commercial or financial relationships that could be construed as a potential conflict of interest.

## References

[1] Chou K-L Liang K Sareen J. The association between social isolation and DSM-IV mood, anxiety, and substance use disorders: wave 2 of the National Epidemiologic Survey on Alcohol and Related Conditions. J Clin Psychiatry. (2011) 72:1468–76. 10.4088/JCP.10m06019gry 21295001

[2] Frucht MM Quigg M Schwaner C Fountain NB. Distribution of seizure precipitants among epilepsy syndromes. Epilepsia. (2000) 41:1534–9. 10.1111/j.1499-1654.2000.001534.x 11114210

[3] Beck A Steer R Brown G Beck depression inventory-II. San Antonio. (1996) 12–5. 10.1037/t00742-000

[4] Spitzer RL Kroenke K Williams JBW Löwe B. A brief measure for assessing generalized anxiety disorder: the GAD-7. Arch Intern Med. (2006) 166:1092–7. 10.1001/archinte.166.10.1092 16717171

[5] Buysse DJ Reynolds CF Monk TH Berman SR Kupfer DJ. The Pittsburgh Sleep Quality Index: a new instrument for psychiatric practice and research. Psychiatry Res. (1989) 28:193–213. 10.1016/0165-1781(89)90047-4 2748771

[6] Curcio G Tempesta D Scarlata S Marzano C Moroni F Rossini PM. Validity of the Italian version of the Pittsburgh sleep quality index (PSQI). Neurol Sci. (2013) 34:511–9. 10.1007/s10072-012-1085-y 22526760

[7] Neufeld MY Sadeh M Cohn DF Korczyn AD. Stress and epilepsy: the Gulf war experience. Seizure. (1994) 3:135–9. 10.1016/S1059-1311(05)80204-3 8081640

[8] Swinkels WAM Engelsman M Kasteleijn-Nolst Trenité DGA Baal MG De Haan GJ Oosting J. Influence of an evacuation in February 1995 in The Netherlands on the seizure frequency in patients with epilepsy: a controlled study. Epilepsia. (1998) 39:1203–7. 10.1111/j.1528-1157.1998.tb01312.x 9821985

[9] Fisher RS Blum DE DiVentura B Vannest J Hixson JD Moss R. Seizure diaries for clinical research and practice: limitations and future prospects. Epilepsy Behav. (2012) 24:304–10. 10.1016/j.yebeh.2012.04.128 22652423

[10] Alexandre V Monteiro EA Freitas-Lima P Pinto KD Velasco TR Terra VC. Addressing overtreatment in patients with refractory epilepsy at a tertiary referral centre in Brazil. Epileptic Disord. (2011) 13:56. 10.1684/epd.2011.0411 21393097

[11] Perucca E Kwan P. Overtreatment in epilepsy. CNS Drugs. (2005) 19:897–908. 10.2165/00023210-200519110-00001 16268662

[12] Mollayeva T Thurairajah P Burton K Mollayeva S Shapiro CM Colantonio A. The Pittsburgh sleep quality index as a screening tool for sleep dysfunction in clinical and non-clinical samples: a systematic review and meta-analysis. Sleep Med Rev. (2016) 25:52–73. 10.1016/j.smrv.2015.01.009 26163057

[13] Gemignani A Piarulli A Menicucci D Laurino M Rota G Mastorci F. How stressful are 105 days of isolation? Sleep EEG patterns and tonic cortisol in healthy volunteers simulating manned flight to Mars. Int J Psychophysiol. (2014) 93:211–9. 10.1016/j.ijpsycho.2014.04.008 24793641

[14] Tsuno N Besset A Ritchie K. Sleep and depression. J Clin Psychiatry. (2005) 66:1254–69. 10.4088/JCP.v66n1008 16259539

[15] Ramsawh HJ Stein MB Belik S-L Jacobi F Sareen J. Relationship of anxiety disorders, sleep quality, and functional impairment in a community sample. J Psychiatr Res. (2009) 43:926–33. 10.1016/j.jpsychires.2009.01.009 19269650

[16] Khan S Duan P Yao L Hou H. Shiftwork-mediated disruptions of circadian rhythms and sleep homeostasis cause serious health problems. Int J Genomics. (2018) 2018:8576890. 10.1155/2018/8576890 29607311 PMC5828540

[17] Daley JT DeWolfe JL. Sleep, circadian rhythms, and epilepsy. Curr Treat Options Neurol. (2018) 20:47. 10.1007/s11940-018-0534-1 30259254

[18] Pratt KL Mattson RH Weikers NJ Williams R. EEG activation of epileptics following sleep deprivation: a prospective study of 114 cases. Electroencephalogr Clin Neurophysiol. (1968) 24:11–5. 10.1016/0013-4694(68)90061-8 4169743

[19] Manganotti P Bongiovanni LG Fuggetta G Zanette G Fiaschi A. Effects of sleep deprivation on cortical excitability in patients affected by juvenile myoclonic epilepsy: a combined transcranial magnetic stimulation and EEG study. J Neurol Neurosurg Psychiatry. (2006) 77:56–60. 10.1136/jnnp.2004.041137 16361593 PMC2117394

[20] Ribot R Kanner AM. Neurobiologic properties of mood disorders may have an impact on epilepsy: should this motivate neurologists to screen for this psychiatric comorbidity in these patients? Epilepsy Behav. (2019) 98:298–301. 10.1016/j.yebeh.2019.01.026 31182393

[21] Tombini M Assenza G Quintiliani L Ricci L Lanzone J Ulivi M. Depressive symptoms and difficulties in emotion regulation in adult patients with epilepsy: association with quality of life and stigma. Epilepsy Behav. (2020) 107:107073. 10.1016/j.yebeh.2020.107073 32320931

[22] Eastwood MR Stiasny S. Psychiatric disorder, hospital admission, and season. Arch Gen Psychiatry. (1978) 35:769–71. 10.1001/archpsyc.1978.01770300111012 655774

[23] Malek N Heath CA Greene J. A review of medication adherence in people with epilepsy. Acta Neurol Scand. (2017) 135:507–15. 10.1111/ane.12703 27781263

[24] Tombini M Assenza G Quintiliani L Ricci L Lanzone J De Mojà R. Epilepsy-associated stigma from the perspective of people with epilepsy and the community in Italy. Epilepsy Behav. (2019) 98:66–72. 10.1016/j.yebeh.2019.06.026 31299536

[25] Kwan P Arzimanoglou A Berg AT Brodie MJ Hauser WA Mathern G. Definition of drug resistant epilepsy: consensus proposal by the *ad hoc* Task Force of the ILAE Commission on Therapeutic Strategies. Epilepsia. (2010) 51:1069–77. 10.1111/j.1528-1167.2009.02397.x 19889013

[26] Malhi GS Mann JJ. Depression. Lancet. (2018) 392:2299–312. 10.1016/S0140-6736(18)31948-2 30396512

[27] Ruscio AM Hallion LS Lim CCW Aguilar-S Al-hamzawi A Alonso J. Cross-sectional comparison of the epidemiology of DSM-5. JAMA Psychiatry. (2017) 74:465–75. 10.1001/jamapsychiatry.2017.0056 28297020 PMC5594751

[28] Pham T Sauro KM Patten SB Wiebe S Fiest KM Bulloch AGM. The prevalence of anxiety and associated factors in persons with epilepsy. Epilepsia. (2017) 58:e107–10. 10.1111/epi.13817 28597927

[29] Gutter T Callenbach PMC Brouwer OF de Weerd AW. Prevalence of sleep disturbances in people with epilepsy and the impact on quality of life: a survey in secondary care. Seizure. (2019) 69:298–303. 10.1016/j.seizure.2019.04.019 31152984

[30] Madrid-Valero JJ Martínez-Selva JM Ribeiro do Couto B Sánchez-Romera JF Ordoñana JR. Age and gender effects on the prevalence of poor sleep quality in the adult population. Gac Sanit. (2017) 31:18–22. 10.1016/j.gaceta.2016.05.013 27474487

[31] Sander JW. The epidemiology of epilepsy revisited. Curr Opin Neurol. (2003) 16:165–70. 10.1097/01.wco.0000063766.15877.8e 12644744

[32] Holt K Shehata A Strömbäck J Ljungberg E Age and the effects of news media attention and social media use on political interest and participation: do social media function as leveller? Eur J Commun. (2013) 28:19–34. 10.1177/0267323112465369

