# Supplementary material for: Epilepsy Care in the Time of COVID-19 Pandemic in Italy: Risk Factors for Seizure Worsening
Source: Front Neurol. 2020 Jul 3;11:737. doi: 10.3389/fneur.2020.00737 (PMC7350269; doi:10.3389/fneur.2020.00737)
Supplement: Supplementary file 1 [file Table_1.docx]

**Supplementary table 1. Principal component analysis on sleep quality (PSQI).**

|  | PC1 | PC2 | PC3 | PC4 | PC5 | PC6 | PC7 |
| --- | --- | --- | --- | --- | --- | --- | --- |
| C1 | 0.5354281 | -0.223111 | 0.3113801 | -0.0118578 | 0.72117923 | 0.08354347 | 0.19839631 |
| C2 | 0.3313268 | -0.280168 | 0.3982551 | 0.6015953 | -0.5279108 | 0.03164591 | 0.1073121 |
| C3 | 0.4506581 | 0.4947062 | -0.201155 | -0.0187816 | -0.1384317 | 0.68899087 | -0.13223059 |
| C4 | 0.4962631 | 0.4374731 | -0.226062 | 0.01618273 | -0.0890331 | -0.6979788 | 0.125987344 |
| C5 | 0.1668098 | -0.100026 | 0.1310783 | 0.01616466 | 0.07095263 | -0.1675631 | -0.95478517 |
| C6 | 0.2630659 | -0.621162 | -0.732824 | -0.0170640 | -0.0755498 | 0.04373433 | -0.00314991 |
| C7 | 0.2390403 | -0.199413 | 0.3140919 | -0.7979820 | -0.4042009 | -0.0120843 | 0.064347629 |
| Cum.Var.Exp | 44% | 62% | 75% | 81% | 89% | 96% | 100% |

**Legend supplementary table 1.** Principal component analysis helps us to reduce dimensionality of data, in order to evaluate which items are responsible of the variation of the total score in our sample we provide the weight matrix of the principal components of PSQI score, showing how the first 3 components explain 75% of the variance of PSQI score. The sub-items contributing with the heaviest weights to these components are:

C1: Subjective evaluation of sleep quality; C2: Sleep Latency;C3: Sleep duration.
